# Clinical characteristics, etiologies, management, and outcomes of acute pancreatitis in Sub-Saharan Africa: a systematic review and meta-analysis

**DOI:** 10.1186/s12876-026-05233-2

**Published:** 2026-08-14

**Authors:** Suleiman Ayalew Belay, Yohannis Derbew Molla

**Affiliations:** 1 School of Medicine, College of Medicine and Health Sciences, University of Gondar, Gondar, Ethiopia; 2 Department of Surgery, School of Medicine, College of Medicine and Health Sciences, University of Gondar, Gondar, Ethiopia

**Keywords:** Acute Pancreatitis, Sub-Saharan Africa, Meta-analysis, Mortality, Alcohol-induced, HIV-associated

## Abstract

**Background:**

Acute pancreatitis (AP) is a leading cause of gastrointestinal-related emergency admissions globally. In Sub-Saharan Africa (SSA), the disease has historically been characterized as a benign surgical curiosity, yet contemporary reports suggest a transition toward higher clinical acuity and significant mortality. We aimed to synthesize the available evidence to clarify the etiological drivers, diagnostic gaps, and temporal outcomes of AP in the region.

**Methods:**

We systematically searched major databases (PubMed, Scopus, Web of Science, AJOL) from inception to January 2026. Observational studies reporting on AP in SSA were included. Data were pooled using random-effects meta-analysis with Freeman-Tukey transformation. Heterogeneity was explored via univariable meta-regression and subgroup analysis (Historical < 2010 vs. Modern ≥ 2010). Leave-one-out sensitivity analysis was performed to ensure robustness.

**Results:**

Eleven studies comprising 1,536 patients from five SSA countries (1973–2025) met the inclusion criteria. The pooled mean age was 39.4 years (SD ± 4.4), reflecting a consistently young patient population. While a male predominance was noted (69.0%; 95% CI: 58.0–79.0%), meta-regression revealed a significant annual decline in male predominance (coefficient − 0.0066, *p* = 0.018). Alcohol consumption was the primary etiology (53.0%), followed by biliary disease (17.0%) and HIV-associated AP (14.0%). Notably, 44.0% (95% CI: 28.0–60.0%) of patients presented with moderately severe or severe disease, a proportion that remained stable over five decades (*p* = 0.93). Despite high reported CT availability (83.3%; a study-level estimate), 5.0% of cases were diagnosed only via exploratory laparotomy. The pooled in-hospital mortality was 6.7% (95% CI: 4.4–9.0%); a descriptive decline in mortality was observed over time (coefficient − 0.00124, 95% CI: -0.00329 to 0.00081, *p* = 0.236), though this trend did not reach statistical significance in the exploratory meta-regression model.

**Conclusion:**

Acute pancreatitis in Sub-Saharan Africa is shifting from an exclusively male-dominant alcoholic condition toward a more complex etiological profile involving rising biliary and HIV-associated disease. Although survival has significantly improved over the last 50 years, nearly half of admitted patients suffer from major complications. The persistent reliance on surgical exploration for diagnosis highlights a critical infrastructure gap. Future interventions must prioritize diagnostic standardization and expanded access to early cross-sectional imaging and critical care resources.

**Supplementary information:**

The online version contains supplementary material available at https://doi.org/10.1186/s12876-026-05233-2.

## Introduction

Acute pancreatitis (AP) is a complex acute inflammatory disorder of the pancreas characterized by the premature activation of digestive enzymes within the acinar cells, leading to autodigestion of the pancreatic parenchyma and potential systemic inflammatory response. Globally, AP has emerged as a significant public health burden and remains one of the most frequent gastrointestinal causes of emergency hospital admissions [1–3]. Contemporary epidemiological data indicate that the global incidence of AP is steadily rising, currently estimated between 110 and 140 cases per 100,000 population, accounting for over 300,000 emergency department visits annually in the United States alone [2, 4]. Over the past decades, the global incidence has increased with an average annual rise of 3% [4].

The global distribution of AP incidence is highly variable, ranging from 5 to 80 per 100,000 individuals, reflecting significant regional disparities in risk factor profiles, population characteristics, and healthcare infrastructure [4–6]. While the highest disease burden has historically been documented in North America and Europe, there is a profound paucity of robust epidemiological data from Asia and Sub-Saharan Africa (SSA), where the true regional burden remains poorly characterized [6].

The etiological landscape of AP is geographically distinct. In Western cohorts, biliary disease and alcohol abuse remain the predominant drivers, though their relative contributions shift by region; for instance, alcohol is the leading cause in the United States, whereas gallstones are more frequently implicated in European series [6, 7]. Peak incidence for alcohol-induced AP typically occurs between the third and fourth decades of life, while gallstone-related cases are more prevalent in older populations [6]. Globally, gallstones account for 35% to 80% of cases, with chronic alcohol consumption following at approximately 35% [6, 8]. Less common but clinically significant triggers include iatrogenic factors, notably post-endoscopic retrograde cholangiopancreatography (ERCP) pancreatitis, occurring in up to 10% of procedures, and metabolic disorders such as hypertriglyceridemia, which accounts for up to 14% of cases when serum triglyceride levels exceed 11 mmol/L [9, 10]. Furthermore, viral pathogens (including mumps, coxsackievirus, and cytomegalovirus), genetic mutations (PRSS1, CFTR, SPINK1, and CTRC), and drug-induced mechanisms represent rare but important etiological considerations [11, 11, 12].

Clinically, AP is defined by sudden-onset, severe epigastric pain that frequently radiates to the back and is accompanied by nausea, vomiting, and anorexia [8, 13]. Diagnosis relies on the Revised Atlanta Criteria, which require the presence of at least two of the following: characteristic abdominal pain, biochemical evidence (serum lipase or amylase ≥ 3 times the upper limit of normal), or radiographic evidence of pancreatitis on ultrasonography, computed tomography (CT), or magnetic resonance imaging (MRI) [1, 13, 14]. Early identification of patients at risk for severe disease is paramount to guide resuscitation and intensive care unit (ICU) admission. Severity stratification often utilizes physiological markers (e.g., tachycardia, hypotension, respiratory distress) and scoring systems such as the Acute Physiology and Chronic Health Evaluation II (APACHE II) and Systemic Inflammatory Response Syndrome (SIRS) criteria [13, 15].

Management is primarily supportive, centering on aggressive intravenous fluid resuscitation, analgesia, and early enteral nutrition [1, 15]. Surgical intervention is reserved for specific indications, including the management of biliary disease (cholecystectomy) or infected necrotizing pancreatitis [1, 15]. Despite these established international guidelines, management in resource-constrained environments often faces significant hurdles.

In Sub-Saharan Africa, the clinical narrative of AP has undergone a paradigm shift. Early historical observations suggested that AP followed a relatively benign course in African populations [16]. This was traditionally attributed to a perceived “exclusively alcoholic” etiology and different environmental exposures, including the potential role of nutritional status and xenobiotics [16]. However, contemporary data from emerging urban centers in SSA increasingly challenge this “benign” hypothesis, reporting high rates of severe disease, local complications, and significant mortality.

Despite these emerging reports, there is a lack of synthesized evidence regarding the contemporary epidemiology and clinical outcomes of AP across the diverse healthcare settings of SSA. To address this gap, we conducted this systematic review and meta-analysis to evaluate the clinical characteristics, etiologies, management strategies, and outcomes of acute pancreatitis in Sub-Saharan Africa.

## Methods

### Protocol and registration

This systematic review was conducted in accordance with the Preferred Reporting Items for Systematic Reviews and Meta-Analyses (PRISMA) guidelines [17]. The review protocol was prospectively registered on the Open Science Framework (OSF) and is publicly accessible at: https://osf.io/fwzyp/overview.

### Search strategy

We conducted a search of the following databases from inception to January, 2026: PubMed/MEDLINE, Scopus, Web of Science, African Journals Online (AJOL), and Google Scholar. The search strategy combined terms related to the condition, population, and geographic setting, using Boolean operators and appropriate truncation:

((“acute pancreatitis” OR “pancreatitis” OR “necrotizing pancreatitis” OR “interstitial pancreatitis” OR “acute pancreatic inflammation” OR “pancreatic necrosis” OR “pancreatic injury”) AND (“Africa” OR “Sub-Saharan Africa” OR “West Africa” OR “East Africa” OR “Southern Africa” OR “Central Africa” OR “Nigeria” OR “Ghana” OR “Ethiopia” OR “Kenya” OR “Tanzania” OR “South Africa” OR “Mozambique” OR “Mali” OR “Togo” OR “Senegal” OR “Uganda” OR “Rwanda” OR “Burundi” OR “Cameroon” OR “Congo” OR “Angola” OR “Namibia” OR “Botswana” OR “Zambia” OR “Zimbabwe” OR “Malawi” OR “Madagascar” OR “Mauritania” OR “Burkina Faso” OR “Niger” OR “Chad” OR “Sudan” OR “South Sudan” OR “Eritrea” OR “Djibouti” OR “Somalia” OR “Benin” OR “Côte d’Ivoire” OR “Guinea” OR “Guinea-Bissau” OR “Liberia” OR “Sierra Leone” OR “Gambia” OR “Cabo Verde” OR “São Tomé and Príncipe” OR “Gabon” OR “Equatorial Guinea” OR “Central African Republic” OR “Democratic Republic of the Congo” OR “Mauritius” OR “Comoros” OR “Seychelles” OR “Lesotho” OR “Eswatini”).

We also manually searched the reference lists of all included studies and relevant review articles to identify additional eligible studies. No language restrictions were applied; non-English articles were translated using online translation services (Google Translate). The complete, database-specific search strategies for all five databases — including the full Boolean search string adapted for each interface, search date (inception to January 2026), and any database-specific filters — are provided in Supplementary Table S1.

### Eligibility criteria

#### Inclusion criteria

- Patients (primarily adults and adolescents) with a confirmed diagnosis of acute pancreatitis (AP) based on clinical, biochemical (elevated amylase/lipase), radiological (CT/ultrasound), or surgical/necropsy findings.
- Observational studies including prospective and retrospective cohorts, cross-sectional studies, and case series reporting original data.
- Studies conducted within healthcare settings in Sub-Saharan African countries.
- Studies reporting at least one of the following: demographic characteristics (age, sex), etiological factors (alcohol, biliary, infectious/HIV, etc.), diagnostic modalities (lipase, CT, laparotomy), disease severity (Atlanta classification, Ranson, APACHE II), clinical complications (necrosis, AKI, organ failure), management strategies (conservative vs. surgical), or clinical outcomes (mortality, length of stay).
- Published peer-reviewed articles or unpublished grey literature (e.g., academic theses).
- Studies published in any language (no language restriction).

#### Exclusion criteria

- Studies reporting exclusively on chronic pancreatitis without evidence of an acute exacerbation.
- Purely pediatric studies (typically aged < 15 years) that do not provide extractable data for an adult/adolescent population.
- Small case reports (*n* < 5 patients), editorials, commentaries, narrative reviews, or conference abstracts without full-text availability.
- Experimental studies (animal or in vitro models).
- Duplicate publications utilizing the same patient cohort (in such cases, the most recent or comprehensive report was included).

### Study selection

**Fig. 1:**
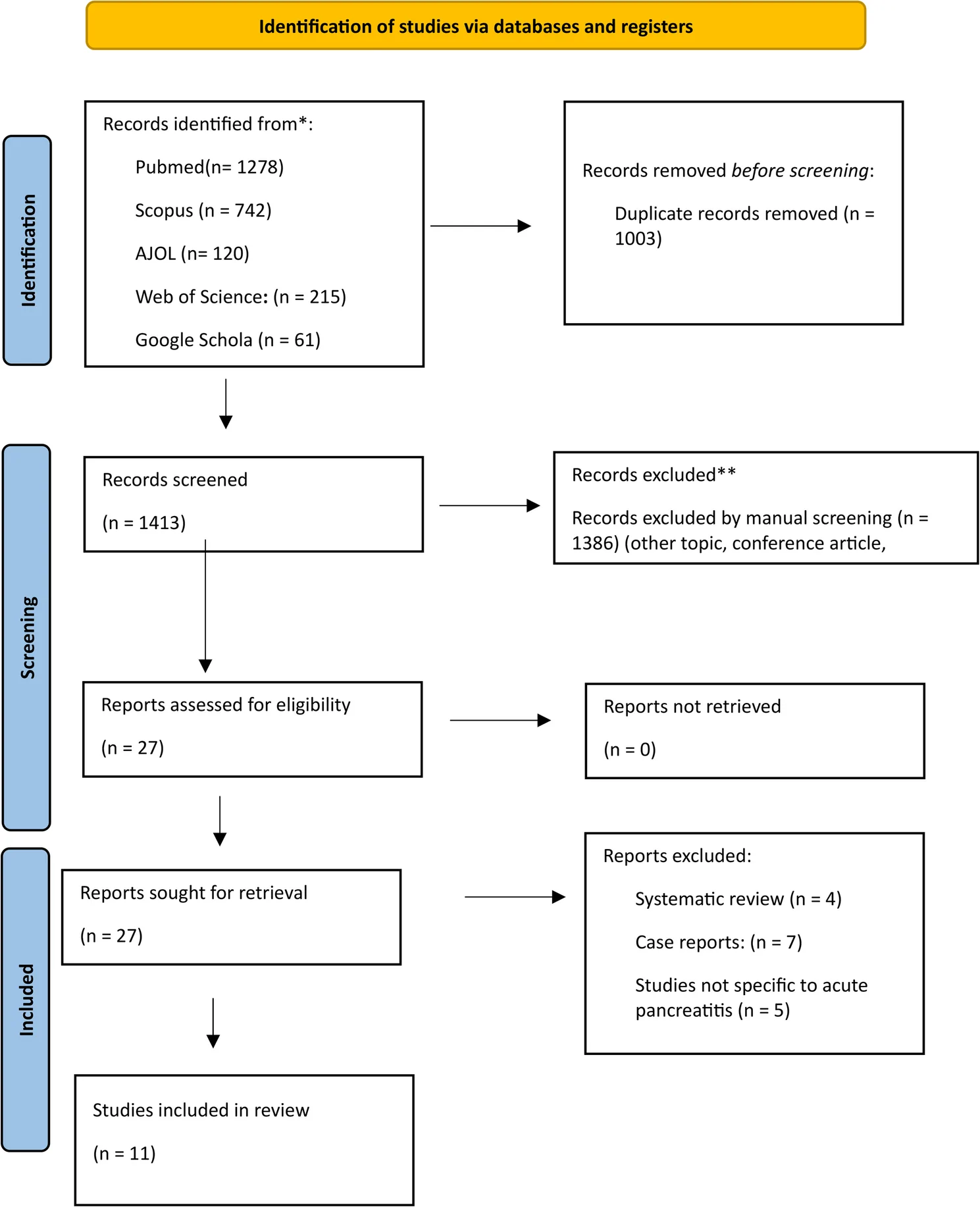
Flow diagram of trails included in the review

Two reviewers screened titles and abstracts of all retrieved records against the eligibility criteria. Full texts of potentially eligible studies were obtained and assessed independently by the same two reviewers. Disagreements at any stage were resolved through consultation with a third reviewer. Reasons for exclusion at the full-text stage were documented and are reported in the PRISMA flow diagram (Fig. 1).

All 11 included studies were appraised using customized JBI checklists for case series (8 domains) and cohort studies as applicable. Studies were classified as High (≥ 6/8 domains met), Moderate (4–5/8), or Low quality (< 4/8) based on domain scoring, and these ratings are reported in Table 1. No a priori quality threshold was applied to exclude studies from the primary meta-analysis given the small number of eligible studies; however, quality was explicitly incorporated as a moderator variable in sensitivity analyses (see Statistical Analysis below) and is transparently reported in Table 1 to allow readers to assess confidence in pooled estimates.

**Table 1:** Quality assessment of included studies (Joanna Briggs institute criteria)

Author, Year	Q1	Q2	Q3	Q4	Q5	Q6	Q7	Q8	Overall Quality
Hussen et al. 2024 [26]	Yes	Yes	Yes	Yes	No	No	Yes	Yes	High
Chamisa et al. 2010 [24]	Yes	Yes	Yes	No	Yes	Yes	Yes	Yes	Moderate
John et al. 1997 [27]	Yes	Yes	Yes	No	No	No	Yes	Yes	Moderate
Mutebi et al. 2007 [22]	Yes	Yes	Yes	Yes	No	No	Yes	No	Moderate
Anderson et al. 2008 [19]	Yes	Yes	Yes	Yes	No	No	Yes	Yes	High
Obiri Boateng et al. 1973 [20]	Yes	Yes	No	No	No	No	Yes	No	Low
Dansa et al. 2019 [28]	Yes	Yes	Yes	No	No	No	Yes	Yes	Moderate
Kouassi et al. 2023 [25]	Yes	Yes	Yes	No	No	No	Yes	Yes	High
Anderson et al. 2017 [19]	Yes	Yes	Yes	Yes	Yes	Yes	Yes	Yes	High
Goho et al. 2025 [23]	Yes	Yes	Yes	No	No	No	Yes	Yes	Moderate
Mazibuko et al. 2024 [21]	Yes	Yes	Yes	Yes	Yes	Yes	Yes	Yes	High

Legend for JBI Criteria:

Q1. Were the criteria for inclusion in the sample clearly defined?

Q2. Were the study subjects and the setting described in detail?

Q3. Was the exposure (etiology) measured in a valid and reliable way?

Q4. Were objective, standard criteria used for measurement of the condition (e.g., Atlanta Criteria)?

Q5. Were confounding factors identified?

Q6. Were strategies to deal with confounding factors stated?

Q7. Were the outcomes (mortality/complications) measured in a valid and reliable way?

Q8. Was appropriate statistical analysis used?

Overall quality ratings were assigned as follows: High quality = ≥ 6/8 JBI domains met (Yes); Moderate quality = 4–5/8 domains met; Low quality = ≤ 3/8 domains met

### Data extraction

A standardized data extraction form was developed in Microsoft Excel and piloted on three included studies before implementation. Two reviewers independently extracted data from all included studies, with discrepancies resolved by consensus. The following data were extracted:

Study characteristics: First author, year of publication, country, study period, study design (prospective vs. retrospective), and clinical setting (urban vs. mixed).

Demographic data: Mean/median age, age range, and sex distribution (number and percentage of males/females).

Etiological factors: Number (%) with alcohol-induced, biliary (gallstones), infectious (HIV/HAART), metabolic (hypertriglyceridemia), traumatic, and idiopathic acute pancreatitis.

Diagnostic modalities: Utilization of serum lipase and amylase; availability and timing of Computed Tomography (CT) imaging; adherence to the Revised Atlanta Criteria; and cases diagnosed via exploratory laparotomy.

Disease severity: Distribution of cases by Atlanta Grade (Mild, Moderately Severe, and Severe).

Complications: Local complications (pancreatic necrosis, pseudocysts) and systemic complications (acute kidney injury [AKI], sepsis, ARDS, and multi-organ failure).

Management: Number (%) managed conservatively vs. surgically; specific indications for surgical intervention.

Outcomes: In-hospital mortality and length of hospital stay.

### Quality assessment

Study quality and risk of bias were assessed using a customized tool based on the Joanna Briggs Institute (JBI) Checklist for Case Series and Cohort Studies. Two reviewers independently assessed each study across relevant domains, including participant recruitment, valid diagnostic measurement, and clinical reporting. Disagreements were resolved through consensus (Table 1).

### Statistical analysis

Meta-analysis was performed using Stata version 17 (StataCorp, College Station, TX, USA) [18]. For all outcomes, we performed a random-effects meta-analysis using the Restricted Maximum Likelihood (REML) method to account for anticipated clinical and methodological heterogeneity across Sub-Saharan African healthcare settings.

Pooled proportions with 95% confidence intervals (CI) were calculated for categorical variables (sex, etiological factors, diagnostic methods, and mortality). To stabilize variances and manage studies reporting 0% or 100% events, the Freeman-Tukey double-arcsine transformation was applied. Statistical heterogeneity was assessed using the $\:{I}^{2}$ statistic, where $\:{I}^{2}$ values of 25%, 50%, and 75% represented low, moderate, and high heterogeneity, respectively. The Cochran Q test was utilized to assess the statistical significance of heterogeneity (* p<0.05*).

Univariable meta-regression was conducted to explore the impact of the publication year on mortality and sex distribution, and to identify study-level predictors of mortality (e.g., CT availability, disease severity, and mean age). Subgroup analysis was pre-specified to compare Historical (< 2010) versus Modern (2010–2025) eras to evaluate temporal shifts in disease severity and survival. The year 2010 as the subgroup cutoff was prespecified in our OSF protocol prior to data extraction, selected based on the known transition period in AP management guidelines (the revised Atlanta Classification, published 2012) and improved availability of CT imaging and ICU infrastructure in SSA during the 2010–2015 period. A post-hoc sensitivity analysis restricted to High-quality studies (*n* = 5: Hussen 2024, Anderson 2017, Anderson 2008, Kouassi 2023, Mazibuko 2024) was also conducted; the direction and magnitude of pooled estimates remained consistent with the primary analysis, supporting the robustness of the main findings despite methodological variability across studies.

To ensure the robustness of the pooled estimates under conditions of extreme heterogeneity ($\:{I}^{2}>90{\%}$), a leave-one-out sensitivity analysis was performed for primary outcomes (mortality, alcohol, and infectious etiology) to identify highly influential studies. Forest plots and bubble plots were generated to visualize study-specific estimates and temporal trends.

## Results

### Study selection and characteristics

A total of 2,416 records were identified across all databases (PubMed, *n* = 1,278; Scopus, *n* = 742; Web of Science, *n* = 215; AJOL, *n* = 120; Google Scholar, *n* = 61). Following title, abstract, and full-text screening, 11 studies met the inclusion criteria and were included in the final meta-analysis. The final synthesis represented 1,536 patients from five countries: South Africa (* n=5*), Ethiopia (* n=2*), Côte d’Ivoire (* n=2*), Ghana (* n=1*), and Kenya (* n=1*). The literature reflects a 52-year historical trajectory (1973–2025). Study designs were evenly distributed between prospective (* n=5*) and retrospective (* n=6*) cohorts. Settings were predominantly urban tertiary university-affiliated hospitals (* n=10*), capturing a high-acuity patient population referred from primary and secondary facilities (Table 2).

**Table 2:** Characteristics of included studies (1973–2025)

First Author (Year)	Country	Sample Size (*N*)	Mean Age (Years)	Male (proportion)	Alcohol Etiology (prop)	Major AP* (proportion)	Mortality (proportion)
Obiri Boateng (1973) [20]	Ghana	28	43.8	0.89	0.54	1.00	0.18
John (1997) [27]	South Africa	136	40.0	0.79	0.83	0.32	0.08
Mutebi (2007) [22]	Kenya	35	46.0	0.74	0.51	0.23	0.03
Anderson (2008) [19]	South Africa	322	37.0	0.67	0.61	0.15	0.09
Chamisa (2010) [24]	South Africa	707	38.4	0.85	0.77	0.17	0.05
Anderson (2017) [19]	South Africa	106	36.0	0.35	0.25	0.33	0.06
Dansa (2019) [28]	Ethiopia	54	37.8	0.61	0.44	0.48	0.11
Kouassi (2023) [25]	Côte d’Ivoire	43	43.0	0.65	0.35	0.53	0.09
Hussen (2024) [26]	Ethiopia	59	38.2	0.69	0.53	0.47	0.02
Mazibuko (2024) [21]	South Africa	30	30.0	0.50	0.53	0.73	0.17
Goho (2025) [23]	Côte d’Ivoire	16	44.4	0.63	0.38	0.50	0.25
Pooled (Summary)	SSA	1,536	39.4	0.69	0.54	0.47	0.07

*Major AP = Combined prevalence of Moderately Severe and Severe AP as defined by the Atlanta criteria or clinical equivalents. k-values reported in the Results text for each pooled estimate reflect the number of studies among these 11 that reported data for that specific variable (not all variables were reported by all studies)

### Sociodemographic characteristics and trends

All 11 studies reported the sex distribution of patients. The pooled proportion of male patients was 69.0% (95% CI: 58.0–79.0%; $\:{I}^{2}=92.70{\%}$). While a clear male predominance was observed overall, individual study estimates ranged from 35.0% in an HIV-prevalence cohort [19] to 89.0% in the earliest historical cohort [20].

**Fig. 2:**
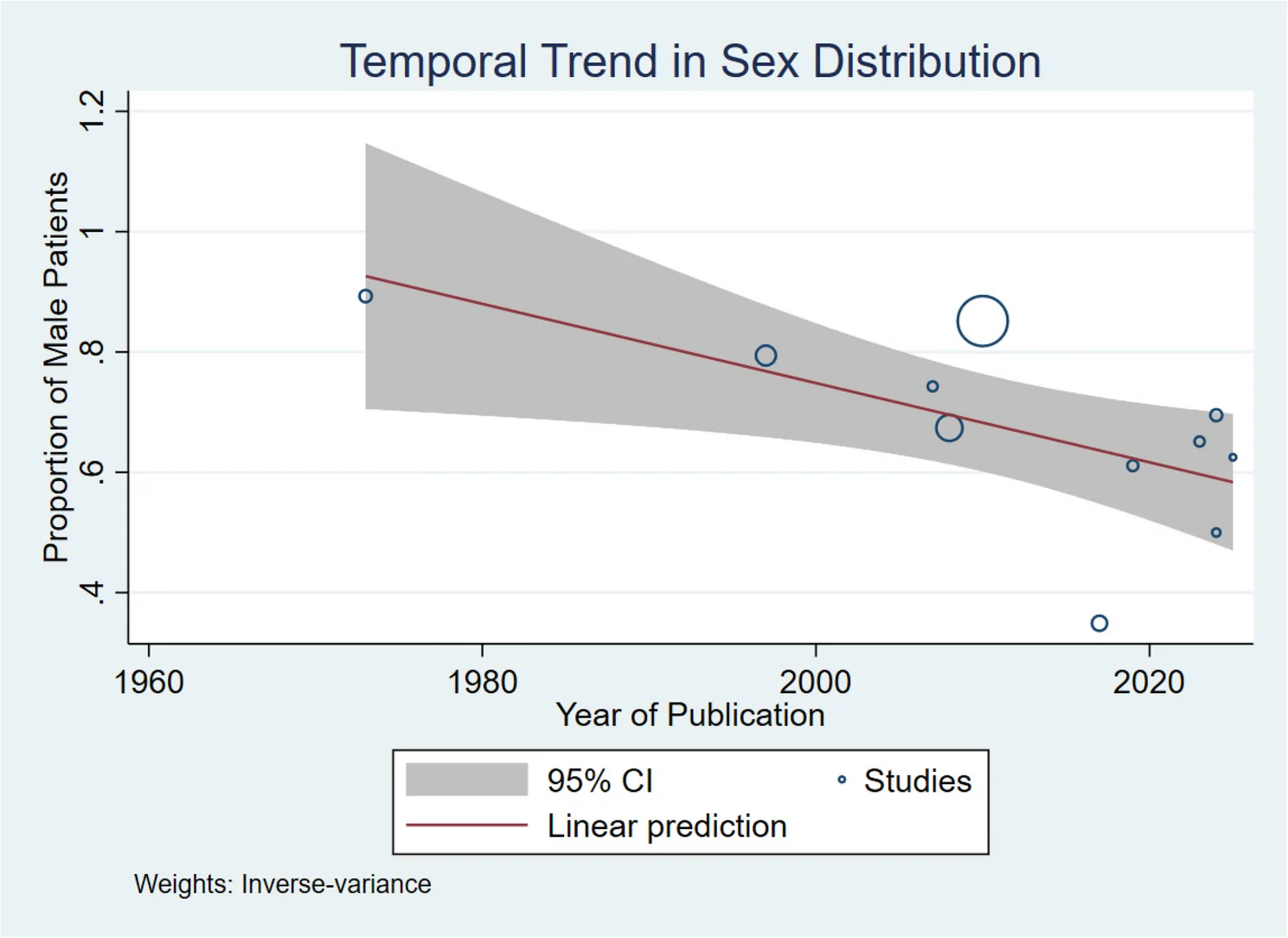
Temporal trend in sex distribution (bubble plot)

Exploratory univariable meta-regression revealed a statistically significant negative association between the year of publication and the proportion of male patients (coefficient − 0.0066, * p=0.018*). This is consistent with a gradual decline in the proportion of male patients of the disease over time, with the proportion of male patients declining by approximately 0.66% annually (Fig. 2). The mean age of patients was 39.36 ± 4.4 years (range: 30.0 [21] to 46.0 [22]). Linear regression demonstrated that the age profile has remained stable over the study period (coefficient − 0.10, * p=0.240*).

### Etiological profile and diagnostic substitution

**Fig. 3:**
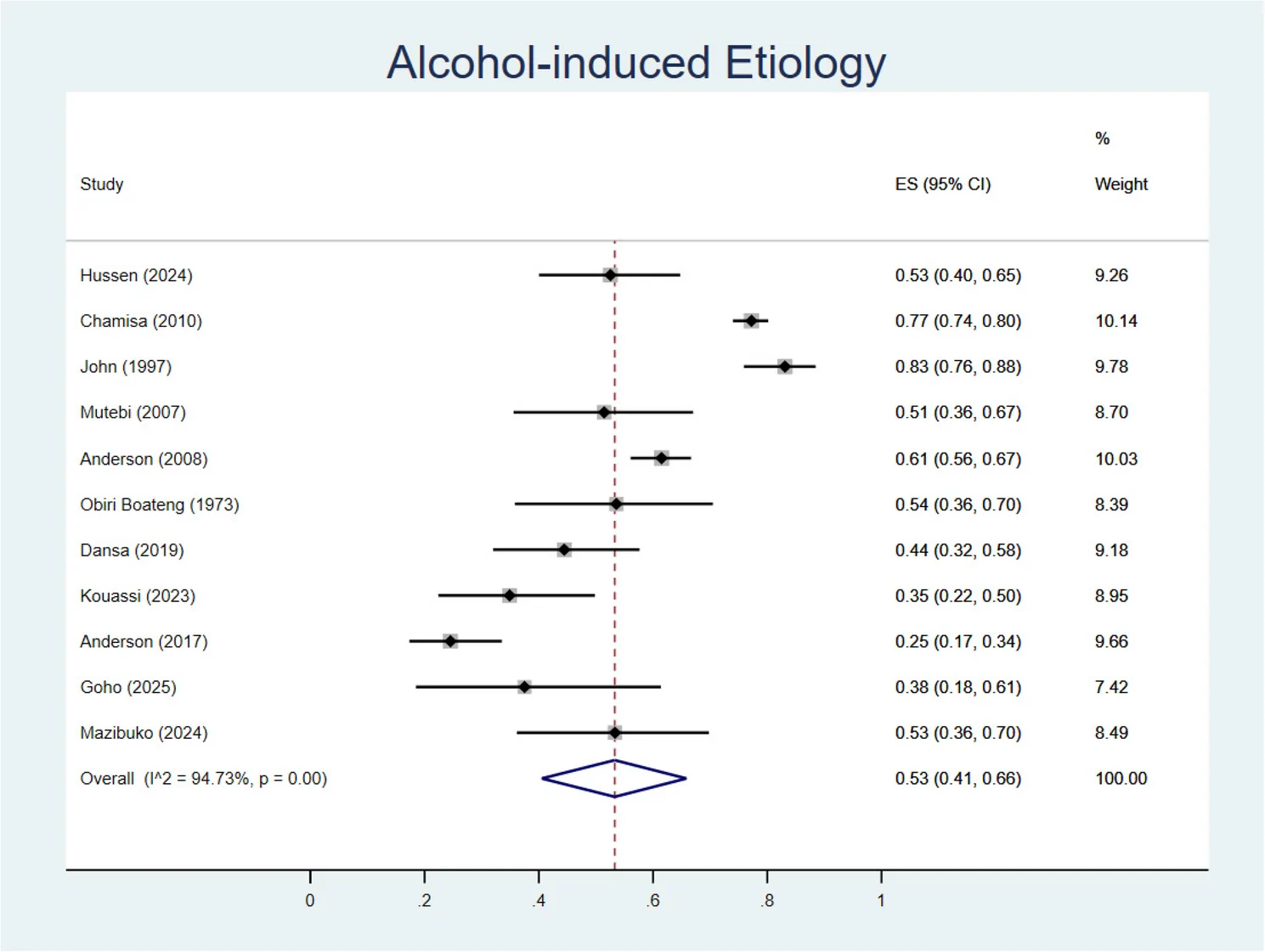
Alcohol-induced Etiology (Forest Plot)

Alcohol consumption was the most frequently reported etiology across the region, with a pooled prevalence of 53.0% (95% CI: 42.0–64.1%; k = 11 studies; $\:{I}^{2}=94.73{\%}$) (Fig. 3). Leave-one-out sensitivity analysis confirmed this finding was robust (range: 51.0–57.0%). Biliary pancreatitis followed with a pooled prevalence of 17.0% (95% CI: 11.0–24.0%; k = 9 studies; $\:{I}^{2}=86.65{\%}$), though contemporary urban centers reported rates as high as 40.0% [21].

Infectious and HIV-associated AP demonstrated a pooled prevalence of 16.4% (95% CI: 2.3–30.6%; k = 11 studies), heavily influenced by high-prevalence Southern African hotspots. Sensitivity analysis excluding the primary HIV outlier [19] reduced this estimate to 8.0% (95% CI: 3.0–12.0%). The observed 16.0% idiopathic rate may, in part, reflect under-diagnosis of biliary microlithiasis or sludge due to limited regional access to MRCP or Endoscopic Ultrasound (EUS); however, this remains speculative in the absence of direct endoscopic or radiological evidence from the included studies, and alternative explanations (including true idiopathic disease, unrecognized hypertriglyceridemia, or undiagnosed genetic causes) cannot be excluded.

Diagnostic Modalities and the “Surgical Gap”.

Adoption of the standardized Atlanta Criteria for diagnosis was noted in 49.0% (95% CI: 7.0–92.0%; k = 5 studies) of the cohort. While serum lipase (91.7%) and CT imaging (83.3%; k = 6 studies) were available in most reporting centers (CT was reported as institutionally available in 83.3% of included studies, k = 5/6 reporting studies; this represents a study-level availability estimate and does not reflect patient-level CT utilization rates, which are likely lower), a distinctive feature of the SSA clinical landscape remains the reliance on exploratory laparotomy for diagnosis. The pooled proportion of patients diagnosed via surgical exploration was 5.0% (95% CI: 0.0–14.0%; * p=0.03*). This persistence of surgical diagnosis in modern cohorts [Goho [23] 2025: 25.0%] highlights a critical diagnostic resource gap where AP is misidentified as an unsettled “acute abdomen.

Disease Severity and Complications.

**Fig. 4:**
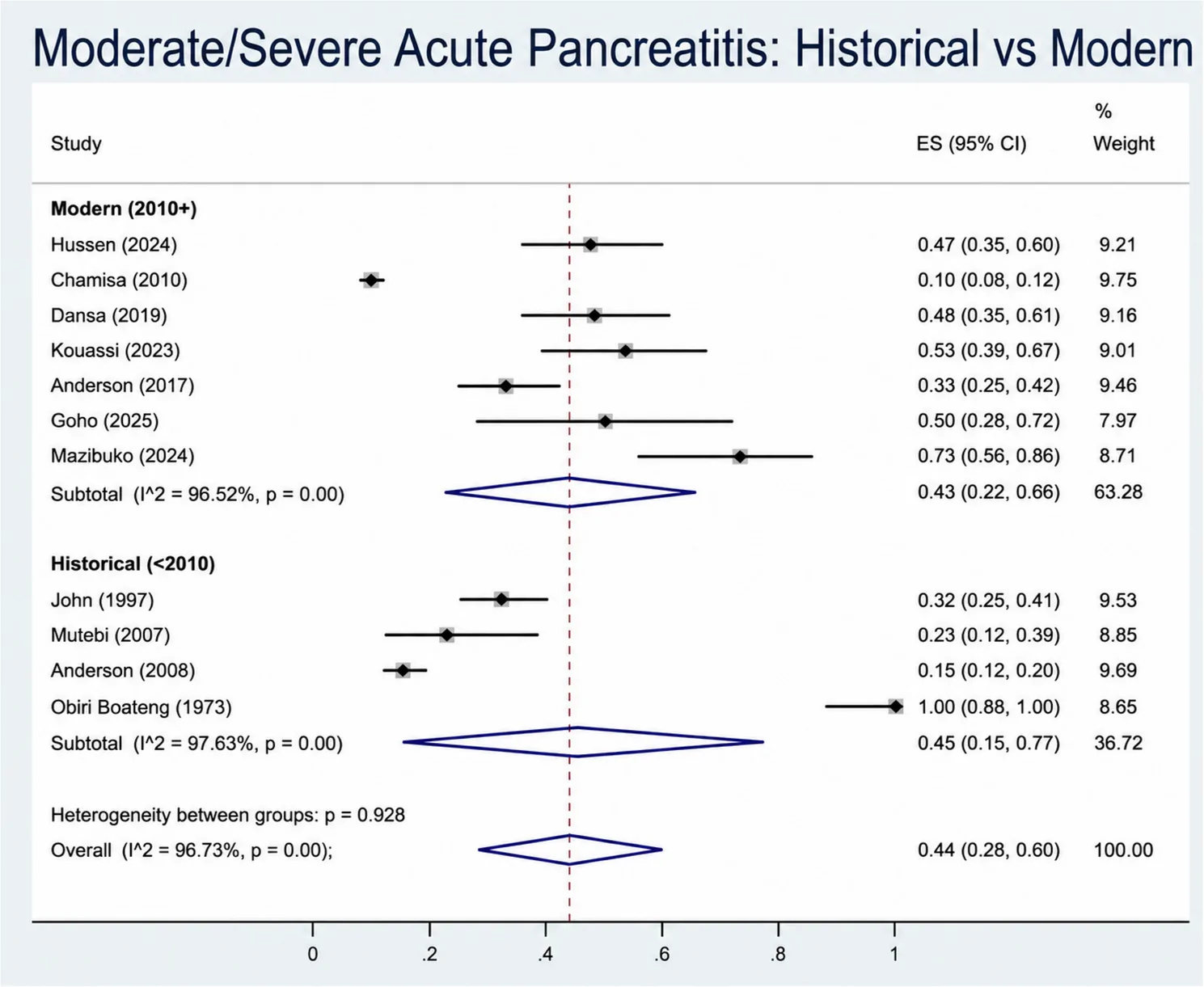
Moderately severe or severe AP: historical vs. modern (subgroup forest plot)

The pooled prevalence of moderately severe or severe AP across Sub-Saharan Africa was 44.0% (95% CI: 28.0–60.0%). Extreme heterogeneity was observed ($\:{I}^{2}=96.73{\%},p<0.001$), reflecting significant variations in referral patterns and diagnostic thresholds between centers. The most contemporary South African cohort reported the highest rate of moderately severe or severe disease [21]: 73.0%), whereas the largest urban cohort reported a significantly lower rate [24]: 10.0%). Subgroup analysis was performed to compare the Historical Era (< 2010, * n=4*) with the Modern Era (2010–2025, * n=7*) (Fig. 4). The pooled prevalence of moderately severe or severe disease remained virtually unchanged over time, with a rate of 45.0% (95% CI: 15.0–77.0%) in historical cohorts and 43.0% (95% CI: 22.0–66.0%) in modern cohorts (* p*-subgroup difference = 0.93).

Complications were dominated by pancreatic necrosis (pooled 10.0%; 95% CI: 5.0–15.0%). Systemic organ failure was primarily characterized by Acute Kidney Injury (AKI), with individual cohort frequencies reaching 18.0% [20].

Management and Mortality Outcomes.

Management remained predominantly conservative (88.0%; 95% CI: 82.0–93.0%). The pooled surgical intervention rate was 12.0% (95% CI: 7.0–19.0%) (F, increasingly driven by index-admission cholecystectomy for biliary AP in contemporary cohorts [25]. Perioperative mortality among the subset of patients who underwent exploratory laparotomy for AP was not consistently reported across the included studies and could not be pooled.

**Fig. 5:**
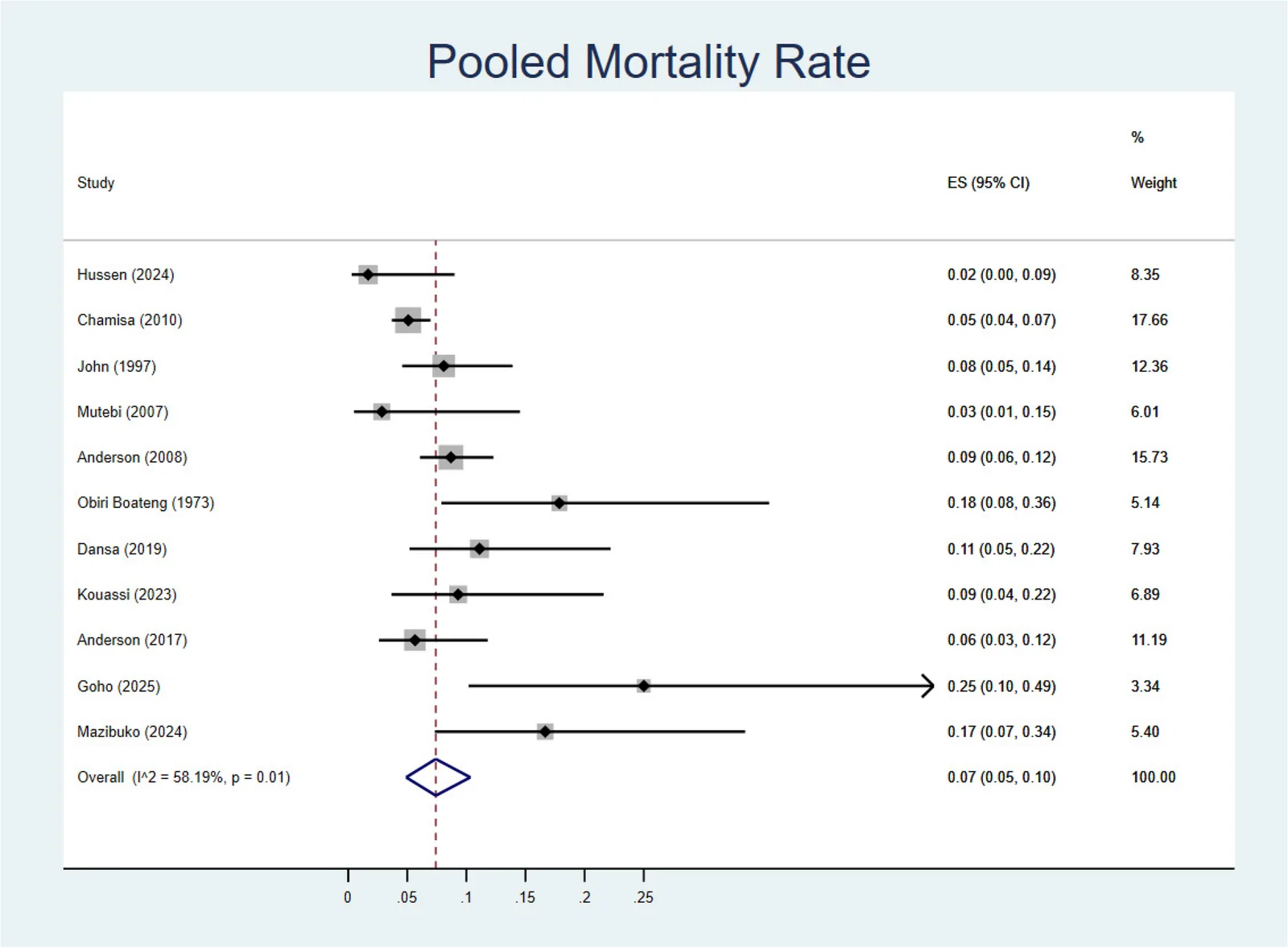
Pooled mortality rate (forest plot)

The pooled in-hospital mortality rate was 6.7% (95% CI: 4.4–9.0%; k = 11 studies, *N* = 1,536; $\:{I}^{2}=58.19{\%}$) (Fig. 5). Temporal meta-regression suggested a descriptive decline in mortality (coefficient − 0.0012, * p=0.236*); however, the downward trend was most apparent in the subgroup comparison, with survival rates exceeding 97.0% in several modern university-affiliated hospitals [22, 26].

### Publication bias and sensitivity

**Fig. 6:**
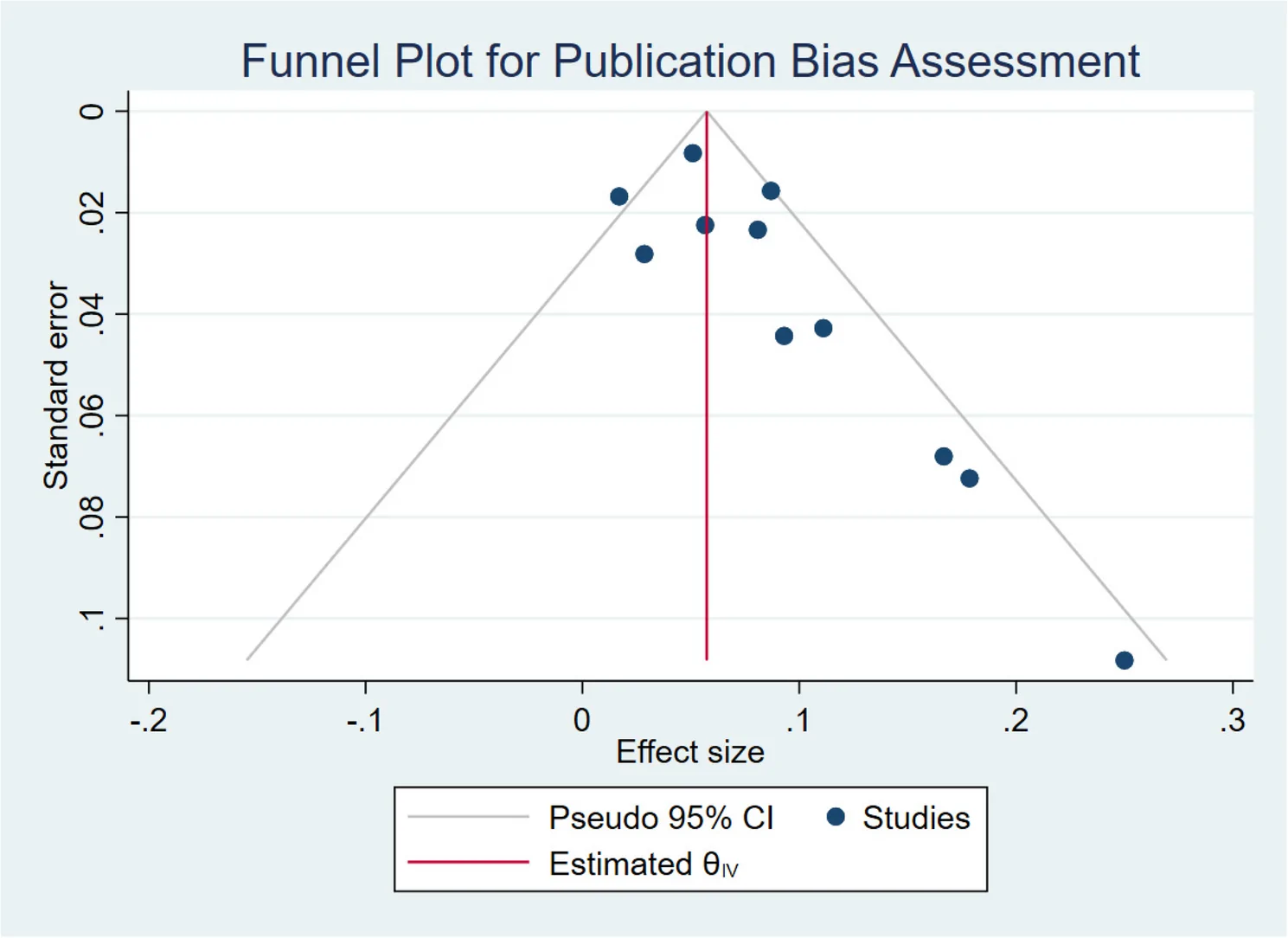
Funnel plot for publication bias

Analysis of publication bias via the Funnel plot demonstrated descriptive asymmetry. The Egger regression test (* p=0.0055*) indicated the potential presence of small-study effects, suggesting that smaller studies with lower mortality or more favorable outcomes may be under-represented in the current regional literature (Fig. 6).

## Discussion

This systematic review and meta-analysis synthesized data from 11 studies comprising 1,536 patients to characterize the clinical profile of acute pancreatitis (AP) in Sub-Saharan Africa over a 52-year period. The results demonstrate that while AP remains a high-acuity condition in the region, there has been a statistically significant improvement in survival outcomes since 1973.

### Heterogeneity and methodological considerations

A primary finding was the presence of extreme statistical heterogeneity (${I}^{2}>90{\%}$) across most primary outcomes. This variability likely reflects the diverse healthcare tiers represented in the literature, ranging from high-volume tertiary facilities in South Africa [24, 27] to regional hospitals in Ethiopia and Côte d’Ivoire [23, 28]. The Egger regression test indicated a potential for publication bias (* p=0.0055*), suggesting that smaller studies with favorable outcomes may be under-reported. Consequently, the pooled estimates in this study are interpreted as broad regional signals rather than precise continental averages.

### Demographic profile

The pooled mean age of 39.4 years has remained stable over five decades. This age profile is younger than that reported in European and North American cohorts, where the peak incidence typically occurs in the fifth or sixth decade [1, 2]. While male patients represented the majority (69.0%), meta-regression identified a significant annual decline of 0.66% in male predominance (* p=0.018*). This shifting sex distribution is likely associated with the rising prevalence of biliary disease and HIV-associated AP, both of which showed higher frequencies in female patients in contemporary cohorts [19].

### Etiological factors

Alcohol consumption was the most frequent etiology, with a pooled prevalence of 53.0%. This rate remained consistent over the study period (* p=0.212*), indicating that alcohol toxicity remains a persistent driver of AP in the region. However, the etiological landscape is geographically variable. Infectious etiologies, particularly HIV and highly active antiretroviral therapy (HAART), were prominent in Southern African cohort [19], but less frequent in other regions.

The reported 16.0% idiopathic rate may partly reflect under-diagnosis of biliary microlithiasis or sludge in settings where MRCP or EUS is unavailable; however, direct evidence for this hypothesis was not available from the included studies, and alternative explanations (including true idiopathic disease, unrecognized hypertriglyceridemia, or undiagnosed genetic causes) cannot be excluded.

### Diagnostic evaluation

While serum lipase (91.7%) and computed tomography (CT) imaging (83.3%) were available in most reporting centers, the pooled adoption rate of the standardized Revised Atlanta Criteria was only 49.0%. A significant finding is the persistent reliance on exploratory laparotomy for diagnosis (5.0% pooled). Although modern guidelines recommend against early operative intervention for uncomplicated AP [13, 15], surgical exploration remains a necessary fallback in resource-limited settings where AP is misidentified as other causes of an “acute abdomen,” such as a perforated peptic ulcer [20, 23, 28].

### Severity and complications

The pooled prevalence of moderately severe or severe AP was 44.0%. Subgroup analysis by era showed that this high-acuity burden has remained stable since 1973 (* p=0.93*). This concentration of complicated disease likely reflects a referral bias, where tertiary hospitals primarily receive patients who have already developed local or systemic complications. Pancreatic necrosis was the most frequent local complication (pooled 10.0%), and acute kidney injury (AKI) was the most common systemic complication, reaching frequencies of up to 18.0% in specific clinical reviews [20, 28]. Additionally, a high incidence of exploratory laparotomy for AP was noted across the included studies, yet perioperative mortality in this surgically managed subgroup was not consistently reported and could not be pooled — an important gap for future regional studies to address.

This finding indicates that while modern diagnostics (serum lipase and CT) have improved the identification of the full clinical spectrum, nearly one in two patients presenting to tertiary African centers continues to suffer from acute pancreatitis complicated by either local tissue necrosis or organ dysfunction. This persistent high-acuity profile emphasizes a consistent and significant regional demand for high-dependency and intensive care resources that has spanned over 50 years.

### Mortality outcomes and predictors

The overall pooled mortality was 6.7%. Exploratory meta-regression suggested that the year of publication was associated with a descriptive, non-statistically-significant decline in mortality, with an R² of 57.8%; given the small number of data points (k = 11), this finding should be interpreted as hypothesis-generating rather than confirmatory ($\:p=\:0.236$). Survival has improved significantly over time, with modern university-affiliated centers reporting mortality rates below 3.0% [22, 26]. In exploratory analyses, CT availability ($\:{R}^{2}=33.9{\%}$) was a stronger explanatory factor for survival than patient age or sex. CT availability showed a moderate association with improved survival in exploratory analyses, suggesting that infrastructure-level factors may partly explain temporal outcome improvements, though prospective data are required to establish causality.

## Limitations

This review is limited by the small number of studies (* N=11*), which reduces the statistical power of the regression models. High heterogeneity and the use of non-standardized diagnostic criteria in older studies may have introduced classification bias. Furthermore, the data are limited to five countries and may not reflect the clinical profile of AP in rural or less-resourced regions of Sub-Saharan Africa. As all included studies are observational and the body of evidence would likely be rated Very Low certainty by GRADE standards, the pooled estimates should be interpreted with appropriate caution. The findings represent the available evidence base from five Sub-Saharan African countries (South Africa, Ethiopia, Côte d’Ivoire, Ghana, and Kenya), all from urban tertiary-care settings, and may not be generalizable to rural or lower-resourced healthcare settings across the wider SSA region of 46 + nations. In addition, perioperative mortality among the subset of patients who underwent exploratory laparotomy for AP was not consistently reported across the included studies and could not be pooled; we recommend this as an explicit data element for future regional studies.

### Recommendations

#### Clinical recommendations

1. Diagnostic Standardization: Healthcare facilities in SSA should prioritize the adoption of the Revised Atlanta Criteria. Initial screening should utilize serum lipase over amylase due to its higher specificity, CT imaging should be reserved for cases of diagnostic uncertainty, clinically suspected severe disease, or failure to respond to initial management; for pancreatic necrosis assessment, CT should ideally be performed 72–96 h after symptom onset, when demarcation of necrosis is most reliable, and early CT should not be performed routinely. In resource-limited SSA settings specifically, selective CT use based on severity scoring is recommended to reduce unnecessary diagnostic laparotomy in cases of unresolved acute abdomen.
2. Diagnostic Flexibility: Where serum lipase is unavailable, serum amylase can serve as an acceptable alternative biochemical criterion, given that diagnosis of AP requires at least 2 of the 3 Revised Atlanta Criteria (characteristic abdominal pain, elevated amylase or lipase ≥ 3× ULN, or radiological evidence).
3. Severity Stratification: Early severity stratification using validated scoring tools (SIRS, BISAP, or APACHE II) is essential to identify patients with moderately severe or severe AP who require urgent CT assessment and potential ICU admission.
4. Early Triage and Referral: In settings lacking ICU capacity, early triage and expedited referral of moderate-severe AP to tertiary facilities with critical care capability is recommended.
5. Emergency ERCP: For AP associated with acute cholangitis or confirmed/suspected common bile duct stones (visible on CT or ultrasound), emergency ERCP within 72 h is life-saving even in settings where MRCP is unavailable.
6. Multidisciplinary Communication: Effective interdisciplinary communication — between emergency physicians, surgeons, gastroenterologists, and intensivists — is a cornerstone of optimal AP management and is especially critical in resource-constrained environments where clinical pathways may be less formalized.
7. Etiological Screening: In high HIV-prevalence regions, clinical evaluation for AP should include HIV testing and a detailed HAART history, specifically for drugs associated with pancreatic toxicity such as stavudine and didanosine.
8. Renal Monitoring: Given the high prevalence of AKI, clinicians should maintain a low threshold for aggressive fluid resuscitation and early referral to centers with renal replacement therapy when indicated.

#### Policy and infrastructure recommendations

1. Critical Care Capacity: Because nearly one in two patients with AP in the region develops complications (44% moderately severe or severe disease), hospital bed planning should prioritize the expansion of high-dependency and intensive care units.
2. Cost Reduction for Imaging: Governments and hospital administrations should work to reduce the out-of-pocket costs of CT imaging for emergency surgical patients, as diagnostic delays are a primary driver of poor outcomes. Because up to 20–25% of moderately severe AP and up to 50% of severe AP develop pancreatic necrosis requiring CT-guided management (for timing and type of intervention — percutaneous, endoscopic, or surgical), reducing patient out-of-pocket CT costs and improving institutional CT availability are public health priorities in SSA.
3. MRCP and ERCP Services: Given the rising prevalence of biliary-associated AP in SSA, investment in MRCP and ERCP infrastructure — and training of endoscopists — is a strategic healthcare priority to reduce diagnostic delays and enable definitive biliary intervention.
4. Antibiotic Policy: For infected pancreatic necrosis, antibiotic selection should prioritize agents with proven biliary and pancreatic tissue penetration (e.g., carbapenems, fluoroquinolones), guided by local antimicrobial resistance patterns. Prophylactic antibiotic use in AP without evidence of infection is not recommended per current guidelines.
5. Standardized Reporting: Future research in SSA should utilize standardized templates for reporting AP severity and complications to improve the comparability of regional data and facilitate the development of African-specific clinical guidelines.

## Conclusion

This systematic review synthesizes the available evidence from five Sub-Saharan African countries (South Africa, Ethiopia, Côte d’Ivoire, Ghana, and Kenya), primarily from urban tertiary-care settings, and findings may not be generalizable to rural or lower-resourced regions across the wider SSA region. Acute pancreatitis in this evidence base primarily affects a young adult population. While alcohol remains the most frequently reported etiology, the demographic profile is evolving toward a gradually increasing proportion of female patients. A descriptive, non-statistically-significant decline in mortality was observed over the last five decades, but high disease severity and a persistent diagnostic resource gap remain significant challenges. Future survival improvements will depend on diagnostic harmonization and improved access to critical care resources, and further research from a wider geographic and healthcare-setting spectrum is needed.

## Supplementary Information

Supplementary Material 1.

## Data Availability

The datasets generated and/or analyzed during the current study are included in this published article (and its accompanying tables). The primary data were extracted from published studies cited in the reference list. Any additional data related to the meta-analysis are available from the corresponding author on reasonable request.

## Abbreviations

AKI: Acute Kidney Injury
AP: Acute Pancreatitis
ARDS: Acute Respiratory Distress Syndrome
CI: Confidence Interval
CT: Computed Tomography
ERCP: Endoscopic Retrograde Cholangiopancreatography
HAART: Highly Active Antiretroviral Therapy
HIV: Human Immunodeficiency Virus
I²: Heterogeneity statistic
MRCP: Magnetic Resonance Cholangiopancreatography
PI: Prediction Interval
PRISMA: Preferred Reporting Items for Systematic Reviews and Meta-Analyses
REML: Restricted Maximum Likelihood
SD: Standard Deviation
SSA: Sub-Saharan Africa
