# Supplementary material for: Clinical characteristics, etiologies, management, and outcomes of acute pancreatitis in Sub-Saharan Africa: a systematic review and meta-analysis
Source: BMC Gastroenterol. 2026 Aug 14;26:754. doi: 10.1186/s12876-026-05233-2 (PMC13756949; doi:10.1186/s12876-026-05233-2)
Supplement: Supplementary file 1 [file 12876_2026_5233_MOESM1_ESM.docx]

**Supplementary Table S1. Database-specific search strategies used for the systematic review**

| **Database** | **Date searched** | **Coverage** | **Search strategy** | **Filters/limits** |
| --- | --- | --- | --- | --- |
| **PubMed/MEDLINE** | January 2026 | Database inception – January 2026 | (("acute pancreatitis"[Title/Abstract] OR pancreatitis[Title/Abstract] OR "necrotizing pancreatitis"[Title/Abstract] OR "interstitial pancreatitis"[Title/Abstract] OR "acute pancreatic inflammation"[Title/Abstract] OR "pancreatic necrosis"[Title/Abstract] OR "pancreatic injury"[Title/Abstract]) AND ("Africa"[Mesh] OR Africa[Title/Abstract] OR "Sub-Saharan Africa"[Title/Abstract] OR "West Africa"[Title/Abstract] OR "East Africa"[Title/Abstract] OR "Southern Africa"[Title/Abstract] OR "Central Africa"[Title/Abstract] OR Nigeria[Title/Abstract] OR Ghana[Title/Abstract] OR Ethiopia[Title/Abstract] OR Kenya[Title/Abstract] OR Tanzania[Title/Abstract] OR "South Africa"[Title/Abstract] OR Mozambique[Title/Abstract] OR Mali[Title/Abstract] OR Togo[Title/Abstract] OR Senegal[Title/Abstract] OR Uganda[Title/Abstract] OR Rwanda[Title/Abstract] OR Burundi[Title/Abstract] OR Cameroon[Title/Abstract] OR Congo[Title/Abstract] OR Angola[Title/Abstract] OR Namibia[Title/Abstract] OR Botswana[Title/Abstract] OR Zambia[Title/Abstract] OR Zimbabwe[Title/Abstract] OR Malawi[Title/Abstract] OR Madagascar[Title/Abstract] OR Mauritania[Title/Abstract] OR "Burkina Faso"[Title/Abstract] OR Niger[Title/Abstract] OR Chad[Title/Abstract] OR Sudan[Title/Abstract] OR "South Sudan"[Title/Abstract] OR Eritrea[Title/Abstract] OR Djibouti[Title/Abstract] OR Somalia[Title/Abstract] OR Benin[Title/Abstract] OR "Côte d'Ivoire"[Title/Abstract] OR Guinea[Title/Abstract] OR "Guinea-Bissau"[Title/Abstract] OR Liberia[Title/Abstract] OR "Sierra Leone"[Title/Abstract] OR Gambia[Title/Abstract] OR "Cabo Verde"[Title/Abstract] OR "São Tomé and Príncipe"[Title/Abstract] OR Gabon[Title/Abstract] OR "Equatorial Guinea"[Title/Abstract] OR "Central African Republic"[Title/Abstract] OR "Democratic Republic of the Congo"[Title/Abstract] OR Mauritius[Title/Abstract] OR Comoros[Title/Abstract] OR Seychelles[Title/Abstract] OR Lesotho[Title/Abstract] OR Eswatini[Title/Abstract])) | No language restriction; Humans |
| **Scopus** | January 2026 | Inception – January 2026 | TITLE-ABS-KEY ("acute pancreatitis" OR pancreatitis OR "necrotizing pancreatitis" OR "interstitial pancreatitis" OR "acute pancreatic inflammation" OR "pancreatic necrosis" OR "pancreatic injury") AND TITLE-ABS-KEY ("Africa" OR "Sub-Saharan Africa" OR "West Africa" OR "East Africa" OR "Southern Africa" OR "Central Africa" OR Nigeria OR Ghana OR Ethiopia OR Kenya OR Tanzania OR "South Africa" OR Mozambique OR Mali OR Togo OR Senegal OR Uganda OR Rwanda OR Burundi OR Cameroon OR Congo OR Angola OR Namibia OR Botswana OR Zambia OR Zimbabwe OR Malawi OR Madagascar OR Mauritania OR "Burkina Faso" OR Niger OR Chad OR Sudan OR "South Sudan" OR Eritrea OR Djibouti OR Somalia OR Benin OR "Côte d'Ivoire" OR Guinea OR "Guinea-Bissau" OR Liberia OR "Sierra Leone" OR Gambia OR "Cabo Verde" OR "São Tomé and Príncipe" OR Gabon OR "Equatorial Guinea" OR "Central African Republic" OR "Democratic Republic of the Congo" OR Mauritius OR Comoros OR Seychelles OR Lesotho OR Eswatini) | No language or document type restrictions during search |
| **Web of Science Core Collection** | January 2026 | Inception – January 2026 | TS=("acute pancreatitis" OR pancreatitis OR "necrotizing pancreatitis" OR "interstitial pancreatitis" OR "acute pancreatic inflammation" OR "pancreatic necrosis" OR "pancreatic injury") AND TS=("Africa" OR "Sub-Saharan Africa" OR "West Africa" OR "East Africa" OR "Southern Africa" OR "Central Africa" OR Nigeria OR Ghana OR Ethiopia OR Kenya OR Tanzania OR "South Africa" OR Mozambique OR Mali OR Togo OR Senegal OR Uganda OR Rwanda OR Burundi OR Cameroon OR Congo OR Angola OR Namibia OR Botswana OR Zambia OR Zimbabwe OR Malawi OR Madagascar OR Mauritania OR "Burkina Faso" OR Niger OR Chad OR Sudan OR "South Sudan" OR Eritrea OR Djibouti OR Somalia OR Benin OR "Côte d'Ivoire" OR Guinea OR "Guinea-Bissau" OR Liberia OR "Sierra Leone" OR Gambia OR "Cabo Verde" OR "São Tomé and Príncipe" OR Gabon OR "Equatorial Guinea" OR "Central African Republic" OR "Democratic Republic of the Congo" OR Mauritius OR Comoros OR Seychelles OR Lesotho OR Eswatini) | No restrictions |
| **African Journals Online (AJOL)** | January 2026 | Inception – January 2026 | "acute pancreatitis" OR pancreatitis AND Africa OR "Sub-Saharan Africa" OR Nigeria OR Ghana OR Ethiopia OR Kenya OR South Africa (adapted to AJOL search interface using combinations of disease and country keywords because Boolean functionality is limited) | No filters applied |
| **Google Scholar** | January 2026 | Inception – January 2026 | "acute pancreatitis" AND (Africa OR "Sub-Saharan Africa" OR Ethiopia OR Kenya OR Ghana OR Nigeria OR "South Africa") Additional searches using "acute pancreatitis" "Sub-Saharan Africa" and individual country names were performed. The first 200 most relevant records were screened in accordance with systematic review recommendations. | Sorted by relevance; first 200 records screened; no language restriction |

**Abbreviations:** AJOL, African Journals Online; TS, Topic Search; TITLE-ABS-KEY, Title/Abstract/Keywords.

**Notes:**

- Searches covered database inception through **January 2026**.
- No language restrictions were applied.
- Reference lists of all included studies and relevant reviews were manually searched for additional eligible studies.
- Grey literature was also considered where available.
- Non-English articles were translated using Google Translate before eligibility assessment.
- Duplicate records were removed before title and abstract screening.
